# The effects of the dopamine stabilizer (−)-OSU6162 on aggressive and sexual behavior in rodents

**DOI:** 10.1038/tp.2016.12

**Published:** 2016-03-22

**Authors:** E Studer, J Näslund, A Westman, A Carlsson, E Eriksson

**Affiliations:** 1 Department of Pharmacology, Institute of Neuroscience and Physiology, Sahlgrenska Academy, University of Gothenburg, Gothenburg, Sweden

## Abstract

The dopamine stabilizer (−)-OSU61612 dampens locomotion in rodents rendered hyperactive by exposure to a novel environment or treatment with amphetamine, but stimulates locomotion in habituated animals displaying low motor activity, tentatively exerting this profile by selectively blocking extrasynaptic D2 receptors. The major aim of the present study was to explore the possible usefulness of (−)-OSU61612 as an anti-aggressive drug. To this end, the effect of (−)-OSU61612 on isolation-induced aggression in male mice and estrous cycle-dependent aggression in female rats were studied using the resident intruder test; in addition, the possible influence of (−)-OSU61612 on sexual behavior in male mice and on elevated plus maze (EPM) performance in male rats were assessed. (−)-OSU61612 at doses influencing neither locomotion nor sexual activity reduced aggression in male mice. The effect was observed also in serotonin-depleted animals and is hence probably not caused by the antagonism of serotonin receptors displayed by the drug; refuting the possibility that it is due to 5-HT1B activation, it was also not counteracted by isamoltane. (−)-OSU61612 did not display the profile of an anxiogenic or anxiolytic drug in the EPM but caused a general reduction in activity that is well in line with the previous finding that it reduces exploratory behavior of non-habituated animals. In line with the observations in males, (−)-OSU61612 reduced estrus cycle-related aggression in female Wistar rats, a tentative animal model of premenstrual dysphoria. By stabilizing dopaminergic transmission, (−)-OSU61612 may prove useful as a well-tolerated treatment of various forms of aggression and irritability.

## Introduction

For a number of psychiatric conditions, symptoms such as violent aggression, anger and irritability contribute considerably to the burden of disease. Patients with mental retardation, brain injury, dementia, stroke and autism may thus all display pathological anger and hostility, and such symptoms are sometimes prominent also in schizophrenia, mania and posttraumatic stress disorder.^1^ In premenstrual dysphoric disorder, irritability is the most frequent complaint,^2^ and it is common also in atypical depression. Although some types of aggression-like behavior may be dampened by antipsychotic drugs,^3^ selective serotonin reuptake inhibitors,^2^ testosterone antagonists or mood stabilizers,^4^ insufficient efficacy and/or cumbersome side effects of all the options available makes the treatment of pathological anger and irritability an important unmet medical need.

Supporting the notion that dopamine exerts a pro-aggressive effect,^5^ drugs eliciting enhanced activation of D2 receptors, including the dopamine releasing-agent amphetamine, the dopamine precursor DOPA and dopamine D2 receptor agonists, may elicit aggression both in experimental animals and humans.^6, 7^ In the same vein, D2 antagonists exert an anti-aggressive effect in rodents,^8^ which is well in line with the observation that both first- and second-generation antipsychotics may dampen aggression in various psychiatric conditions.^3^ The cumbersome side effects marring both traditional and atypical antipsychotic drugs, however, make these drugs far from optimal for this purpose.

The phenylpiperidine (−)-OSU61612, displaying affinity but no or weak intrinsic activity for dopaminergic D2 receptors *in vivo*,^9^ modulates D2 receptor-influenced behavior differently as compared with all previously known D2 antagonists.^10, 11^ Hence, although (−)-OSU61612 mimics the effect of D2 antagonists by dampening locomotion in animals displaying hyperactivity after having been placed in a novel environment^11, 12^ or treated with amphetamine,^9, 12^ it increases locomotion in rats with low activity; because of this profile, it has been named a dopamine stabilizer.^9, 13, 14^ Though displaying an antipsychotic-like profile in various tentative animal models of schizophrenia,^12, 15^ unlike most D2-blocking antipsychotic drugs (−)-OSU61612 does not cause catalepsy even at high doses in rodents;^12^ in line with this, early clinical experience suggests (−)-OSU61612 to be largely devoid of the extrapyramidal side effects characterizing most D2-blocking antipsychotics.^16^

Although the mechanisms underlying the unique profile of (−)-OSU61612 remains to be clarified, one explanation might be that it is a D2 antagonist with fast dissociation properties acting predominantly on extrasynaptic D2 receptors, including both D2 receptors serving as autoreceptors, brain D2 receptors situated extrasynaptically at non-dopaminergic neurons and prolactin-regulating D2 receptors in the pituitary.^11, 14, 17^ In line with this, PET studies suggest (−)-OSU61612 to antagonize only a subset of D2 receptors in the human striatum.^18^ Other mechanisms may, however, also be of importance for its unique behavioral profile, such as an alleged allosteric influence on the D2 receptor; the literature on this possibility is, however, conflicting.^10, 11^ An involvement of serotonin receptors in the mechanism of action of (−)-OSU61612 should also not be excluded as it displays affinity for at least three of these, the 5-HT2A, 5-HT2C and 5-HT1B receptors.^10^

A compound that is able to dampen the activation of D2 receptors in a situation of enhanced dopaminergic activity without interfering with normal locomotion, and without displaying the metabolic side effects caused by most atypical antipsychotics, would be of potential use as an aggression-dampening drug. The major aim of the present study hence was to explore the possible influence of (−)-OSU61612 on aggression and other social behaviors in isolated male mice and on estrus cycle-related aggression in females (a tentative animal model of premenstrual dysphoria) using the resident intruder (RI) paradigm. In addition, it was studied whether the anti-aggressive effect of (−)-OSU61612 might be explained by interference with serotonergic receptors, and also to what extent the drug, at doses corresponding to those exerting an anti-aggressive effect, influences sexual behavior and exploration in a situation associated with avoidance, that is, the elevated plus maze (EPM), respectively.

## Materials and methods

### Animals

For tests of the effect of (−)-OSU61612 on male aggressive behavior (experiments I, II and III), sexual behavior (experiment IV) and locomotion (experiment V), CD-1 mice aged 10–16 weeks were used. In the mouse RI experiments (experiments I, II and III), male mice of the 129Sv/Ev strain^19^ aged 11–13 weeks served as intruders. For the exploration of male sexual behavior (experiment IV), gonadectomized 18–20-week-old C57Bl/6 females were used as stimulus mice. Effects of (−)-OSU61612 on EPM performance (experiment VI) was studied in 12-week-old male Wistar rats. For assessing the effect of (−)-OSU61612 on estrus cycle-dependent aggression in females (experiment VII), Wistar rats aged 14 weeks were used as residents and female Wistar rats aged 10 weeks as intruders. The animals were allowed at least 1 week of acclimatization before the experiments and kept under controlled conditions with 12/12 light/dark cycle (lights on at 0600 h) with free availability of food and water. With the exception of the EPM experiments, all the tests were performed during the dark phase under infrared illumination. In all the experiments, the treatment was randomly assigned to individual animals. All the procedures were approved by the Ethical Committee on Animal Experiments, Gothenburg, Sweden.

### Drugs

(−)-OSU6162 ((S)-(−)-(3-methanesulfonyl-phenyl)-1-propyl-piperidine, PNU-96391; kindly donated from Pfizer, New York, NY, USA) and isamoltane hemifumarate (Tocris Bioscience, Bristol, UK) were dissolved in 0.9% saline solution. Para-chlorophenylalanine methyl ester hydrochloride (pCPA; Sigma Aldrich, Stockholm, Sweden) was dissolved in 0.9% saline solution and buffered to a pH around 5.5 with a drop of 0.1 N sodium hydroxide solution. Estradiol benzoate (Sigma Aldrich) and progesterone (Sigma Aldrich) were dissolved in sesame oil.

### Experiment I

After 7 days of individual housing with no change of bedding to allow for the formation of territorial behavior, male CD-1 mice were screened for aggression using the RI test, preceded by 15 min of habituation to a transparent acrylic glass lid used to enable video recording. The test was conducted by introducing a male intruder mouse, weighing at least 10 g less than the resident, in the opposite corner relative to that of the intruder followed by 15 min of video-recorded social interactions.^19^ A purpose of this initial RI test was to discard mice not displaying aggression from further experiments; however, no non-aggressive animals were identified. After eight additional days, during which the animals remained isolated, they were administered (i) (−)-OSU6162 (75 μmol kg^−1^), (ii) (−)-OSU6162 (150 μmol kg^−1^) or (iii) saline 30 min before being re-tested in the RI test with a novel intruder. The recordings were scored at low speed by an observer unaware of the treatment of the animals. All instances of attack bites, chasing of the intruder and threat postures were defined as aggressive behavior, whereas all types of sniffing, following, approach, attending and grooming were defined as social behavior.^20^ Encounters during which the intruder displayed marked aggressive behavior were excluded.

### Experiment II

The influence of serotonin depletion on the effect of (−)-OSU6162 (150 μmol kg^−1^) on aggressive behavior (assessed as in experiment I) in male CD-1 mice was tested. All the animals were housed in individual cages for at least 13 days before the experiment. Starting 3 days before the test, they were given the irreversible serotonin synthesis inhibitor pCPA at a daily dose of 300 mg kg^−1^ (or saline) with the last injection administered 24 h before testing. As in experiment I, the animals were administered (−)-OSU6162 (150 μmol kg^−1^) or saline 30 min before testing, the experimental groups being (i) pCPA plus (−)-OSU6162, (ii) saline plus (−)-OSU6162, (iii) pCPA plus saline or (iv) saline plus saline. The video recordings and exclusion criteria were assessed as in experiment I.

### Experiment III

The possible influence of the 5-HT1B antagonist isamoltane^21, 22^ (5 mg kg^−1^) on the effect of (−)-OSU6162 (150 μmol kg^−1^) on aggressive behavior (assessed as in experiment I) in male CD-1 mice was explored. The animals were administered (−)-OSU6162 (150 μmol kg^−1^) or saline 30 min before testing, the experimental groups being (i) isamoltane followed 15 min later by (−)-OSU6162, (ii) saline followed by (−)-OSU6162, (iii) isamoltane followed by saline or (iv) saline followed by saline. The video recordings and exclusion criteria were assessed as in experiment I.

### Experiment IV

The effect of (−)-OSU6162 (75 μmol kg^−1^ and 150 μmol kg^−1^) on sexual behavior in male CD-1 mice was first tested using sexually naive animals. The mice were individually housed for 48 h after which a gonadectomized female mouse, made sexually receptive by pretreatment with estrogen benzoate 10 μg (−48 h), estrogen 5 μg (−24 h) and progesterone 500 μg (−4 h), was introduced, with interactions being videotaped for 30 min.^23^ The mice that attacked the female were excluded from this and further experiments. Subsequently, the mice obtained further sexual experience for a 3-day period during which they were co-housed with a female mouse primed with estrogen benzoate at doses of 10 and 5 μg of estradiol benzoate 48 and 24 h earlier, respectively; mice that did not mount and those that attacked the female during the first 30 min were excluded from the remainder of the experiment. Three days after removal of the female, the mice were again tested with respect to sexual behavior after treatment with (−)-OSU6162 or saline. In the first test, the sexual performance was assessed by quantifying the attempted mounts (that is, misdirected mounts or the male mounting as the female moved away), completed mounts and mounts with intromission, the latter characterized by slow rhythmic pelvic thrusts while clasping the flank of the female mouse.^24^ In the second test, the three different types of mounts were collapsed and scored as mounting only.

### Experiment V

The aim of experiment V was to assess whether the effects of (−)-OSU6162 on aggressive behavior observed in previous experiments is secondary to a general reduction in locomotion. To make the conditions the same as for the RI experiments, the CD-1 mice were housed individually for 15 days before being tested for locomotion in six sound-attenuated cages (420 × 420 × 200 mm) with a grid of photocell beams across the floor of the cage with beam breaks automatically registered by a computer (Kungsbacka mät- och reglerteknik, Fjärås, Sweden).^25^ The animals were allowed to habituate for 1 h in the box before being given intraperitoneal injections with OSU 75 μmol kg^−1^, OSU 150 μmol kg^−1^ or saline; immediately after drug administration, they were returned to the box and activity recorded as 10-min blocks for a total of 60 min.

### Experiment VI

For assessment of the possible effect of (−)-OSU6162 on explorative and anxiety-like behavior, a previously published protocol was used.^26^ The animals were placed in the center of a standard black acrylic plastic rat EPM (Med Associates, St Albans, VT, USA) with a light level in the center of the maze of 35 lux. Each test lasted for 5 min and was video-recorded for analysis of entries to open arms, closed arms and center, as well time spent on open arms. One hour before testing, the animals had been given intraperitoneal injections of OSU6162 (25 μmol kg^−1^), (−)-OSU6162 (100 μmol kg^−1^), (−)-OSU6162 (200 μmol kg^−1^) or saline.

### Experiment VII

A previous protocol by Ho *et al.*,^27^ assessing estrus cycle-dependent performance in the RI test was applied to study the influence of (−)-OSU6162 on aggression in females. To this end, the female Wistar rats aged 13–14 weeks were housed in groups of three and subjected to assessment of estrus cycle phase by daily examination of the cytology of vaginal smears for at least two cycles; rats not displaying a normal four to five days cycle were discarded. In line with the previous observations, according to which only a subpopulation of female rats display aggressive behavior using this paradigm,^27^ the first RI test, conducted in the diestrus phase, had the purpose of excluding animals not displaying aggression. Before the test, two of the three rats inhabiting the cage were removed; 15 min later, a female intruder aged 10 weeks and weighing at least 25 g less than the resident was introduced for a period of 15 min during which the interactions between the animals were videotaped. As in the previous publication describing this paradigm,^27^ a composite aggression score, modified from Albert *et al.*,^28^ was calculated as follows: composite aggression=(number of attacks)+0.2 × (attack duration, s)+(number of bites)+0.2 × (on-top duration, s). Only those displaying aggression were used for subsequent testing. After this screening test, estrus cycle was again determined daily by vaginal smears; after one or two full cycles, a second RI test, with a novel intruder, was conducted with the animals being in diestrus and having been administered (−)-OSU6162 (75 μmol kg^−1^), (−)-OSU6162 (200 μmol kg^−1^) or saline 30 min earlier.

### Statistics

The effects of treatment for all the tests but locomotion were analyzed using one-way analysis of variance followed by Fisher's least significant difference test. In experiment V, the effects of drug treatment on locomotion were analyzed using two-way (dose × time) analysis of variance followed by Dunnett's multiple comparisons test. With an alpha error rate of 5% and a power of 80%, using an additive effect model in the present dose-response studies (I, IV, V, VI, VII), the standardized mean difference that would be detected for each individual experiment is as follows; experiment I: 0.6, experiment IV: 0.7, experiment V: 0.8, experiment VI: 0.8 and experiment VII: 0.7. Corresponding standardized mean difference for pairwise comparisons in experiment II and III is 0.5 and 0.7, respectively.

## Results

### Experiment I

**Figure 1:**
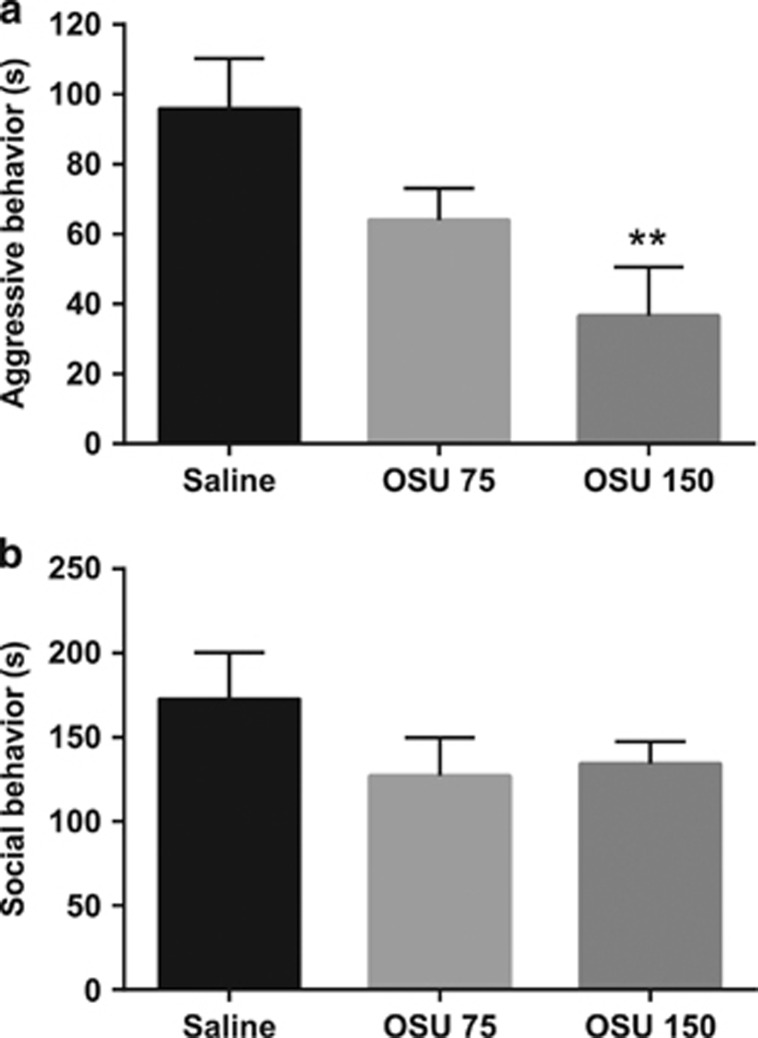
Results from the resident intruder experiments in male mice. (**a**) Duration of aggressive behavior in experiment I, in male mice treated with saline (*n*=13), (−)-OSU6162 (75 μmol kg^−1^; OSU 75, *n*=14) or (−)-OSU6162 (150 μmol kg^−1^; OSU 150, *n*=13). (**b**) Duration of social behavior in experiment I. All the data are presented as mean (±s.e.m.). **P<0.01 versus mice treated with saline.

(−)-OSU6162 induced a dose-dependent reduction of the duration of aggressive behavior (Figure 1a) without influencing the number of mice displaying aggression or the latency to the first aggressive act (data not shown). (−)-OSU6162 did not influence the other aspects of social behavior (Figure 1b).

### Experiment II

Arguing against the possibility of the anti-aggressive effect of (−)-OSU6162 being mediated by antagonism of any serotonergic receptor subtype exerting a pro-aggressive influence (such as the 5-HT2A or 5-HT2C receptor), the effect of (−)-OSU6162 was unabated in mice subjected to inhibition of serotonin production by means of 3 days of pretreatment with the synthesis inhibitor pCPA at a dose known to cause a marked reduction of brain serotonin. The duration of aggression in the different groups were: saline+saline: 145.0±21.0; pCPA+saline: 123.0±19.6 (versus saline+saline: not significant); saline+OSU: 64.8±12.0 (versus saline+saline: *t*(48)=3.25, *P*<0.01); pCPA+OSU: 74.9±15.4 (versus saline+saline: *t*(48)=2.84, *P*<0.01; versus pCPA+saline: *t*(48)=2.05, *P*<0.05; analysis of variance, F(3,48)=4.92, *P*<0.01). One pCPA-treated animal died before testing.

### Experiment III

Arguing against the possibility that the anti-aggressive effect of (−)-OSU6162 is mediated by agonism of the 5-HT1B serotonergic receptor subtype, it was not influenced by pretreatment with the 5-HT1B antagonist isamoltane administered 15 min before (−)-OSU6162. The duration of aggression in the different groups were: saline+saline: 165.0±9.8; isamoltane+saline: 163.0±32.8 (versus saline+saline: not significant); saline+OSU: 62.9±16.0 (versus saline+saline: *t*(25)=3.56, *P*<0.01); isamoltane+OSU: 87.8±14.8 (versus saline+saline: *t*(25)=2.78, *P*<0.05; versus pCPA+saline: *t*(25)=2.70, *P*<0.05; analysis of variance, F(3,25)=6.72, *P*<0.01). Three encounters were excluded from the analysis as the intruder displayed marked aggressive behavior.

### Experiment IV

In sexually naive mice, administration of (−)-OSU6162 did not influence any of the recorded aspects of sexual behavior, that is, latency to mounts, total time of mounts, latency to first intromission or total time of mounts with intromissions (Table 1A); also, there was no effect on the latency to misdirected mounts or total time of misdirected mounts (data not shown). In the second test, after acquisition of sexual experience, there was again no significant effect of (−)-OSU6162 on male sexual behavior (Table 1B). In all, seven mice displayed aggression towards the female during the first test or during the first 30 min of co-housing with a female and were hence removed from the experiment.

### Experiment V

**Figure 2:**
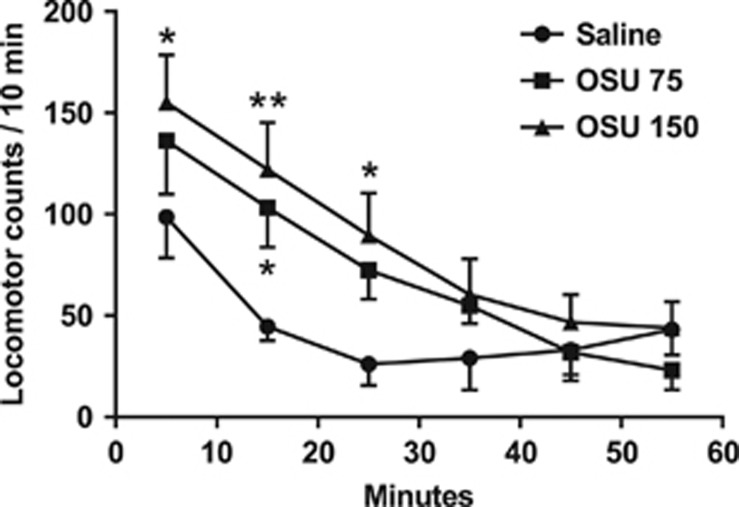
Locomotor activity in habituated mice injected with (−)-OSU6162 (75 μmol kg^−1^; OSU 75, *n*=7), (−)-OSU6162 (150 μmol kg^−1^; OSU 150, *n*=6) or saline (*n*=6). Data are presented as group mean of beam breaks for each 10-min interval±s.e.m. *P<0.05, **P<0.01 versus saline.

Refuting the possibility that (−)-OSU6162 reduces aggressive behavior by inducing a general impairment of locomotor activity, the drug did not reduce locomotion in isolated male mice 30–60 min after drug administration, that is, at the time when an anti-aggressive effect had previously been observed. During the first 30 min post injection, the mice administered 150 μmol kg^−1^ of (−)-OSU6162, on the contrary, displayed an increase in locomotion. The mice receiving the lower dose, 75 μmol kg^−1^, displayed enhanced locomotion during 10–20 min after administration (Figure 2).

### Experiment VI

**Figure 3:**
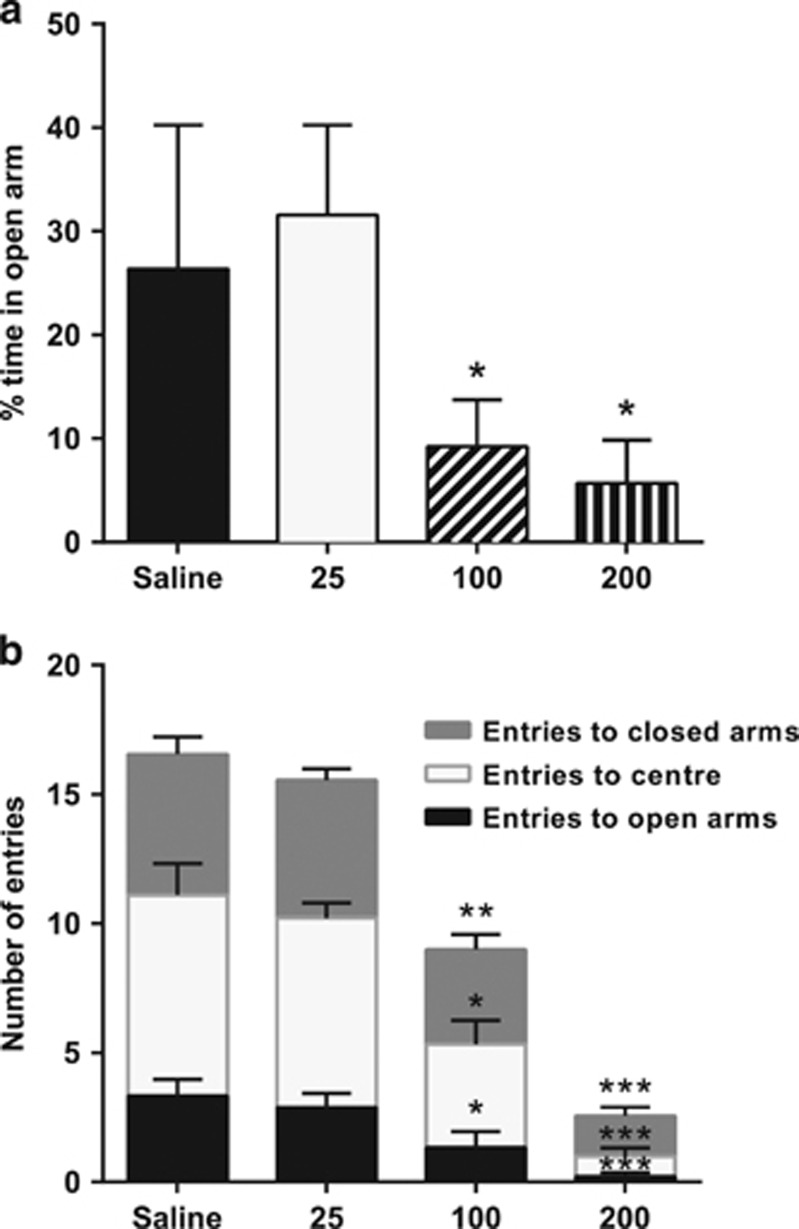
Elevated plus maze behavior of rats treated with saline (*n*=9), (−)-OSU6162 (25 μmol kg^−1^; *n*=9), (−)-OSU6162 (100 μmol kg^−1^; *n*=9), or (−)-OSU6162 (200 μmol kg^−1^; *n*=9). (**a**) Percentage of time spent on the open arms. (**b**) Entries to open arms, closed arms and center. *P<0.05, **P<0.01, ***P<0.001 versus saline.

In the EPM, (−)-OSU6162 exerted a dose-dependent reduction of the time spent on the open arms (Figure 3a) as well as of entries to open arms, closed arms and center (Figure 3b).

### Experiment VII

Of a total of 47 rats tested for aggression in the diestrus phase, 37 displayed aggression in the screening test and were hence used in the subsequent experiment. (−)-OSU6162 (75 μmol kg^−1^) and (−)-OSU6162 (200 μmol kg^−1^) induced a reduction in aggression of similar magnitude while leaving social behavior unaffected (Table 2).

## Discussion

We report that a dopamine stabilizer, (−)-OSU6162, elicits a dose-dependent reduction of aggressive behavior at doses not interfering with the other aspects of social behavior, general locomotor activity or sexual behavior. These observations, in conjunction with early clinical experience showing this compound to be devoid of antipsychotic-like side effects,^16^ suggest that (−)-OSU6162 could prove to be a useful option for the treatment of aggression in humans.

In case the atypical dopamine-stabilizing profile of (−)-OSU6162 is caused by a selective antagonism of extrasynaptic D2 receptors, as there are reasons to believe,^29^ it may be concluded that this pool of D2 receptors is involved in the regulation of isolation-induced aggression, but not of the other aspects of social behavior that were studied in the RI experiments, as these were not impaired by the drug. Likewise, in a separate experiment, (−)-OSU6162 did not reduce locomotion 30–60 min after drug administration, corresponding to the time period they were tested with respect to aggressive behavior. During the first 10–30 min after drug administration, the animals displayed an increase in locomotion, which is well in line with what has previously been found in habituated rats.^12, 13^

Of note is that clozapine and amisulpride,^30^ like (−)-OSU6162, have been reported to reduce aggression in the RI paradigm at doses not interfering with locomotion in general.^31, 32^ With respect to more conventional D2 antagonists, most studies have failed to separate the anti-aggressive effect from a general locomotion-impairing action;^20, 33, 34^ some reports however suggest that one may obtain a reduction of aggression at doses slightly lower than those influencing locomotion.^35^ D2 autoreceptors, which to a greater extent than postsynaptic D2 receptors may be extrasynaptic, are suggested to be targeted by both D2 agonists^36^ and antagonists^37^ at lower doses than those required to influence postsynaptic D2 receptors; the possible aggression-reducing effect of low doses of, for example, amisulpiride^32^ is hence well in line with the hypothesis that the dopaminergic influence on aggression may be at least partly regulated by extrasynaptic D2 receptors.

Though dopaminergic stabilization is the most probable mechanism of action for the anti-aggressive effect of (−)-OSU6162, other possibilities should not be overlooked. In this context, it is of interest to note that the compound is reported to display considerably higher affinity to sigma receptors than to dopamine D2 receptors.^38^ This being the mechanism underlying the anti-aggressive effect, however, appears unlikely given the clear dose-response relationship observed for the anti-aggressive effect in the male mice (but not in the female rats); in case this influence were mediated by sigma antagonism, a maximal effect should have been expected already at the lower dose.

A complex involvement of serotonin in the regulation of rodent aggression is well established; for example, both 5-HT2A^39^ and 5-HT2C^40^ antagonism have been reported to exert an anti-aggressive effect. As (−)-OSU6162 has been shown to display relatively high affinity for the 5-HT2A receptor, and some (but lower) affinity for the 5-HT2C receptor, probably acting as partial agonists at both these subtypes,^10, 13^ we found it justified to address the possibility that 5-HT2A or 5-HT2C antagonism could be the mechanisms underlying its anti-aggressive properties. The observation that the effect of (−)-OSU6162 was unabated in mice being the subject of depletion of brain serotonin by means of pretreatment with the serotonin synthesis inhibitor pCPA, however, renders the hypothesis that (−)-OSU6162 reduces aggression by counteracting a pro-aggressive influence of serotonin mediated by 5-HT2A and/or 5-HT2C receptors unlikely. Likewise, the possibility that its anti-aggressive effect is mediated by activation of the 5-HT1B receptor, which is known to exert an anti-aggressive influence,^41^ and to which (−)-OSU6162 displays some (but low) affinity and high agonist activity,^10^ was refuted by the observation that its influence on aggression was not blocked by pretreatment with isamoltane administered at a dose previously shown to effectively antagonize this receptor subtype.^22^

To further explore the possible behavioral relevance of an influence of (−)-OSU6162 on serotonergic transmission at doses corresponding to those reducing aggression, we also evaluated the possible effect of the drug on rats tested in the EPM, where the propensity to avoid open arms, an alleged proxy of anxiety, is influenced by both 5-HT1B and 5-HT2 receptors.^42, 43^ (−)-OSU6162 did reduce time spent on open arms, but this should probably not be interpreted as an anxiogenic effect as the drug markedly reduced entries not only onto open arms, but also to the center and closed arms, which is well in line with the previous observation that the drug, probably because of its stabilizing influence on dopaminergic neurotransmission, reduces the exploratory behavior of animals placed in a novel environment.^11, 12^

The regulation of sexual behavior and that of aggressive behavior display certain striking similarities. Thus, serotonin exerts largely an inhibitory influence on both sexual behavior and aggression, whereas testosterone and dopamine promotes both;^24, 44^ a reduction in sexual drive hence is a common side effect of many compounds exerting an anti-aggressive effect, such as serotonin reuptake inhibitors and testosterone antagonists. This aspect, as well as the fact that both aggressive and sexual behavior may activate reward circuits,^45, 46^ in conjunction with previous reports suggesting (−)-OSU6162 to counteract both brain stimulation reward^14^ and the rewarding properties of ethanol,^47^ prompted us to explore to what extent the anti-aggressive effect of this compound is associated with a dampening influence also on the sexual behavior. No such effect being observed, however, suggests that the population of D2 receptors regulating male sexual behavior were not targeted by (−)-OSU6162 at the tested doses.

Violent aggression is much more common in men than in women, whereas enhanced irritability and anger in the luteal phase of the menstrual cycle are important symptoms experienced by women afflicted by premenstrual dysphoric disorder.^48^ We have previously shown that a subpopulation of female Wistar rats tested in the RI paradigm displays aggression in the metestrus or diestrus but not the estrus phase of the estrus cycle, and that this behavior, like the irritability characterizing premenstrual dysphoric disorder, may be abolished both by gonadectomy and by the administration of a serotonin reuptake inhibitor.^27^ The observation that (−)-OSU6162 exerts an anti-aggressive effect not only in male mice, but also in this putative animal model of premenstrual dysphoric disorder, suggests that the drug might serve as an alternative to the current first line of treatment for premenstrual dysphoric disorder, that is, the serotonin reuptake inhibitors.^2^

The anatomical position of the tentatively extrasynaptic D2 receptors mediating the anti-aggressive effect of (−)-OSU6162 remains an open question. One area of possible interest in this context is a portion of the anterior hypothalamus^49^ where antagonists of D2 receptors administered locally have been shown to reduce offensive aggression.^50^ Another example of a region that is of importance for the regulation of aggression and which harbor D2 receptors is the nucleus accumbens.^45, 51^

In conclusion, the present data suggest that a dopamine stabilizer, (−)-OSU6162, may exert a specific anti-aggressive effect in both males and females. The compound deserves to be tested for conditions characterized by anger, hostility, irritability and aggression.

## Figures and Tables

**Table 1:** Effects of (−)-OSU6162 on sexual behavior in male mice

*A*					
*Naive*	n	*Mounts without intromission*		*Mounts with intromission*	
		*Duration (s)*	*Latency (s)*	*Duration (s)*	*Latency (s)*
Saline	11	82.5 (±10.9)	561 (±120)	45.2 (±10.8)	1016 (±148)
OSU 75	12	59.0 (±15.2)	950 (±147)	71.2 (±17.2)	1067 (±146)
OSU 150	10	58.6 (±7.8)	667 (±126)	56.8 (±17.4)	1038 (±159)

*B*					
*Experienced*	n	*All mounts*			
		*Duration (s)*	*Latency (s)*		
Saline	11	305.6 (±30.7)	443 (±35)		
OSU 75	12	236.9 (±41.2)	583 (±75)		
OSU 150	10	224.9 (±39.3)	530 (±99)		

Sexual behavior in male mice treated with saline, (−)-OSU6162 (75 μmol kg^−1^; OSU 75) or (−)-OSU6162 (150 μmol kg^−1^; OSU 150). (A) Mounts and mounts with intromission in sexually naive mice. (B) Total mounts (including mounts with intromission) in sexually experienced male mice. Data are presented as mean (±s.e.m.). There were no significant differences between the groups.

**Table 2:** Effects of (−)-OSU6162 on aggressive behavior in female rats

	n	*Aggression composite (±s.e.m.)*	P*-value* vs *saline*	*Social behavior (s) (±s.e.m.)*	P*-value* vs *saline*
Saline	12	30.0 (±5.8)	—	151.5 (±16.5)	—
OSU 75	12	5.8 (±2.5)	0.0001	142.6 (±13.3)	NS
OSU 200	13	5.9 (±2.8)	0.0001	120.0 (±17.5)	NS

Abbreviation: NS, not significant.

Aggressive and social behavior during the resident intruder test in female rats studied in the diestrus phase and pretreated with saline, (−)-OSU6162 (75 μmol kg^−1^; OSU 75), or (−)-OSU6162 (200 μmol kg^−1^; OSU 200). Data are presented as mean (±s.e.m.).
